# Exploring knowledge, attitudes, and preventive practices related to dengue fever control: a cross-sectional study of 1,016 individuals

**DOI:** 10.1080/20477724.2026.2621706

**Published:** 2026-01-24

**Authors:** Anan S. Jarab, Walid Al-Qerem, Ahmad Z Al Meslamani, Zainab Abdelnasser, Omar Jrab, Tareq Mukattash, Maher Khdour, Yazid N Al Hamarneh, Judith Eberhardt

**Affiliations:** a Department of Clinical Pharmacy, Faculty of Pharmacy, Jordan University of Science and Technology, Irbid, Jordan; b Department of Pharmacy, Faculty of Pharmacy, Al-Zaytoonah University of Jordan, Amman, Jordan; c College of Pharmacy, Al Ain University, Abu Dhabi, United Arab Emirates; d Barts and the London School of Medicine and Dentistry, Queen Mary University of London, Victoria, Malta; e Faculty of Pharmacy, Al-Quds University, Jerusalem, Palestine; f Department of Pharmacology, Faculty of Medicine and Dentistry, University of Alberta, Edmonton, Canada; g Department of Psychology, School of Social Sciences, Humanities and Law, Teesside University, Middlesbrough, UK

**Keywords:** Dengue fever, knowledge, attitudes, practices, control

## Abstract

Dengue fever is a growing global health concern, especially in regions like the United Arab Emirates (UAE), where environmental factors and high levels of international travel increase the risk of outbreaks. Despite this, public awareness and adoption of preventive measures remain understudied. This cross-sectional study aimed to assess the public’s knowledge, attitudes, and practices (KAP) regarding dengue fever and its prevention. A validated online questionnaire was distributed to 1106 individuals using convenience sampling. The results revealed significant knowledge gaps, with only 24.6% correctly identifying dengue as a viral infection and 24.5% recognizing mosquito transmission. Median knowledge scores were higher among participants with elementary education (median = 13, IQR: 13-18), postgraduate degrees (median = 12, IQR: 8-15) than among those with other education levels, and higher among those with health insurance (median = 11, IQR: 10-13) than among those without insurance. Attitudes varied significantly based on education and residency, while regression analysis showed that male gender, older age, and smoking were associated with higher practice scores. These findings emphasize the need for targeted educational campaigns and public health interventions to improve awareness and engagement with dengue prevention, particularly in vulnerable demographic groups.

## Introduction

Dengue fever is a viral infection caused by the Dengue virus (DENV), a mosquito-borne virus of the genus *Flavivirus* and the family *Flaviviridae*. Four serotypes of the virus cause dengue: DEN-1, DEN-2, DEN-3, and DEN-4. Infection with one serotype provides lifelong immunity to that specific serotype but only short-term immunity to the other serotypes [1,2]. Dengue is primarily spread to humans through the bites of infected female *Aedes aegypti* mosquitoes and, to a lesser extent, *Aedes albopictus* [2]. These mosquitoes become infected when they bite a person already infected with the dengue virus [2].

Most people with dengue experience mild or no symptoms and recover in 1–2 weeks; however, in rare cases, the disease can be severe and potentially fatal [2,3]. Symptoms of dengue, if they occur, typically begin 4–10 days after infection and last 2–7 days. These can include high fever, severe headache, pain behind the eyes, muscle and joint pains, nausea, vomiting, swollen glands, and rash [3].

The global prevalence of dengue has increased dramatically in recent decades, rising from 505,430 cases in 2000 to 5.2 million in 2019 [4]. In 2023, over 6.5 million cases and more than 7,300 deaths were reported. As of 2024, more than 7.6 million cases have been reported, with over 16,000 severe cases and more than 3,000 deaths. This disease also imposes a considerable financial burden. A comprehensive analysis reported that the total cost of dengue infection in 2022 was approximately 94.7 billion US dollars [5].

Currently, there are two vaccines with proven efficacy against dengue virus: Dengvaxia and QDENGA (TAK-003) and were approved by the WHO [6–8]. However, they are not universally available and have specific usage recommendations. For example, Dengvaxia is approved for use in individuals aged 9–16 who have had a previous dengue infection and live in endemic areas. This vaccine is not recommended for those who have never been infected with dengue due to the risk of severe disease from subsequent infections [7]. Since dengue fever is preventable, effective preventative strategies include following evidence-based guidelines, and adhering to recommended practices.

A global systematic review including 59 studies from the general population found that only about 40 % had sufficient knowledge of dengue, 47 % exhibited positive attitudes, and fewer than 40 % engaged in effective preventive behaviors [9]. Regionally, a systematic review in Thailand reported similarly low knowledge (35 %) and practices (25 %), despite moderately positive attitudes (61 %) [10]. Another systematic review covering evidence from 2010 to 2022 reveals that across Asian households, although knowledge and attitudes were generally acceptable, preventive practices were inconsistent and often inadequate [11]. A recent KAP study from Indonesia showed knowledge at 46 %, attitudes at 65 %, and practices at 56 %; notable gaps persist, especially regarding awareness of dengue serotypes and infection risk [12].

The United Arab Emirates (UAE) is vulnerable to dengue fever due to its status as a major global travel hub and environmental factors conducive to mosquito breeding. The frequent influx of international travelers can introduce the dengue virus, which, coupled with the UAE’s urban environment and climate changes, creates optimal conditions for *Aedes mosquitoes*, the primary vectors of dengue. These conditions include increasing temperatures and fluctuating rainfall patterns [13–15]. Following the UAE’s heaviest recorded rainfall in April 2024 and reports from hospitals of dengue cases without travel history

The campaign’s objectives were to suppress transmission through (i) rapid case detection and immediate electronic reporting; (ii) targeted entomological surveillance with GPS mapping of breeding sites; (iii) elimination of *Aedes aegypti* larval habitats; (iv) laboratory-supported pesticide sensitivity testing; and (v) multi-language public education, coordinated with the Ministry of Climate Change and Environment and municipalities under WHO-aligned integrated vector management. Reported outputs at that time included deployment of nine Emirates Health Services specialist teams, 1,200 entomological surveys, analysis of 309 mosquito DNA samples (with the Abu Dhabi Agriculture & Food Safety Authority), elimination of 409 breeding sites, and readiness of 134 facilities for dengue diagnosis and care. These actions followed warnings and intensified control measures after the April floods and coincided with a documented surge in cases [16].

Since dengue infections have historically been uncommon and frequently linked to importation from other countries, the UAE is not considered a typically endemic area for the disease. For example, there was no evidence of a local vector at the time of the first confirmed dengue case in the UAE, which was described as an imported illness [17]. However, there have been isolated locally acquired cases in recent years, most notably a number of cases in Dubai involving individuals who had never traveled [18]. These reports correlate with exceptionally high rainfall and flooding events, which create ideal conditions for Aedes mosquito hatching [17]. Given that Aedes aegypti’s top larval tolerance range is between approximately 37 and 42 °C, typical UAE temperatures (frequently above 38 °C) might decrease mosquito survival and larval growth [19]. Nevertheless, microhabitats, standing water from flash floods, and sporadic cooler periods may allow for local transmission and limited mosquito persistence. Furthermore, exposure risk and the unequal geographic distribution of KAP survey data are likely influenced by the UAE’s rapid and uneven urbanization, which varies greatly between more rural Emirates such as Umm Al-Quwain and more urbanized Emirates such as Dubai and Abu Dhabi.

Despite the potential health risk dengue poses in the UAE, public awareness and adoption of preventive measures have not previously been assessed. Therefore, this study aimed to examine the public’s knowledge, attitudes, and practices (KAP) regarding dengue fever in the UAE. The findings should inform the development of targeted education campaigns and policy decisions, ultimately enhancing dengue prevention and control efforts and reducing both the incidence and impact of the disease.

## Materials and methods

### Study design and participants

In this cross-sectional survey, a research pharmacist distributed a validated questionnaire through Google Forms on popular social media platforms such as WhatsApp, Instagram, Facebook, and Telegram. The survey targeted individuals over 18 years old across various emirates in the UAE and employed convenience sampling. Data were collected during June and July 2024.

### Ethics approval

The current research received the required ethical approval from the research ethics committee at Al Ain University, Abu-Dhabi Campus (Ref. No. COP/AREC/AD/57). Before completing the study questionnaire, participants were presented with information about the study, and then asked to tick a box to indicate informed consent to participate in the study. No incentives were offered to participants for completing the study questionnaire.

### Sample size calculation

Using the Krejcie & Morgan equation to calculate the minimum required sample size [20], the formula indicated that for an indefinite population size with a 95% significance level, a 5% margin of error, and a 50% population proportion, at least 385 participants were needed.

### Study instrument

The study’s questionnaire was adapted from the World Health Organization’s April 2024 report on dengue fever and other related studies [3,21,22]. An expert panel including two professors in public health and two professors in epidemiology evaluated the comprehensiveness and clarity of the questionnaire. Subsequently, a pharmacy graduate piloted the survey with fifteen individuals to assess item relevance, clarity, and completion time. Based on feedback from this pilot, adjustments were made, including the rephrasing of ambiguous terms, to enhance comprehension. The findings from the pilot were not included in the final data analysis. The Cronbach’s alpha for the knowledge, attitude, and practice scales were 0.73, 0.77, and 0.71, respectively, demonstrating the reliability of the study instrument.

**Figure 1.**
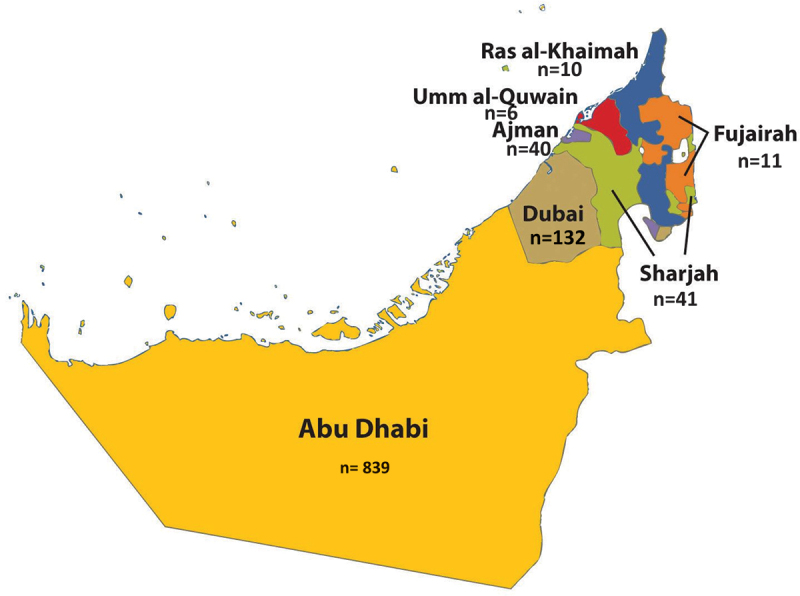
Distribution of the study sample (N=1106).The western region is part of Abu Dhabi.

The questionnaire began with a brief introduction outlining the study’s objectives, emphasizing participant confidentiality and anonymity. The first section collected sociodemographic information, including age, gender, area of residency, marital status, monthly income, education level, employment status and field of work, health insurance, and smoking status. The distribution of the study sample across the UAE is represented in Figure 1 The second part of the questionnaire consisted of 21 items evaluating participants’ knowledge of various aspects of dengue. These included the etiology and nature of the disease, transmission methods, symptoms during and after infection, potential consequences, and supportive, pharmacological, and vaccine-related treatments. Items were structured as yes/no questions, with each ‘yes’ response scoring 1 point. The total knowledge score was calculated by summing all correct answers, with a maximum possible score of 20. The third part of the questionnaire assessed participants’ attitudes towards the disease and its prevention using five items on a 5-point Likert scale ranging from ‘strongly disagree’ (1 point) to ‘strongly agree’ (5 points). The attitude domain comprised five items designed to measure (i) perceived susceptibility to dengue infection, (ii) perceived benefits and effectiveness of source reduction (e.g., removing breeding sites), (iii) perceived effectiveness of personal protection measures (repellent, long sleeves, screens/nets), and (iv) collective responsibility (community participation/clean-ups) and trust in official guidance. Example items included: “Removing mosquito breeding sites around homes reduces the chance of dengue infection,” “Using insect repellent and long-sleeved clothing can protect people from dengue,” “I am at risk of dengue in the UAE,” and “Participating in community clean-ups helps prevent dengue.” Negatively phrased items were reverse-coded so that higher scores uniformly indicate more favorable attitudes toward evidence-based prevention.

.

The final section on practices covered strategies employed by the UAE public to combat the virus, assessed through 18 items constructed as ‘yes’ or ‘no’ questions. It consisted of two subdomains: (a) vector control/personal protection and (b) supportive care during illness. Vector/personal protection items covered: eliminating standing water in and around the household; covering water storage containers; installing/maintaining window and door screens or using bed nets when appropriate; using EPA-registered insect repellents (e.g., DEET, picaridin/icaridin, IR3535) as directed; and wearing long sleeves and trousers outdoors. Supportive-care items assessed behaviors recommended during febrile illness, including adequate oral hydration, using acetaminophen (paracetamol) for fever/pain, avoiding NSAIDs (e.g., ibuprofen, aspirin) due to bleeding risk, seeking medical care for warning signs, and limiting mosquito exposure while ill (e.g., screened/air-conditioned room or bed net). Each “yes” response was coded 1 and “no” 0; counterproductive actions (if any) were reverse-coded. The total practice score was the sum of all items (range 0–18).

### Statistical analysis

The Statistical Package for the Social Sciences (SPSS) version 26 was utilized for data analysis. The Kolmogorov-Smirnov test was applied to assess the normality of continuous variables at once, including knowledge, attitude, and practice scores. The test revealed a p-value of 0.002, indicating a significant deviation from a normal distribution and non-parametric assumptions. Consequently, continuous variables were presented as medians with interquartile ranges (25th to 75th percentiles). Differences in knowledge and attitude scores across sociodemographic variables were assessed using the Mann-Whitney U test and the Kruskal-Wallis H test, as appropriate. The Mann-Whitney U test was used to compare median values between two independent groups, such as male and female participants. In contrast, the Kruskal-Wallis test was used to examine the differences across three or more independent groups, such as comparing median scores by age categories or place of residence. A p-value of less than 0.05 considered statistically significant. Finally, a multiple linear regression model was constructed to explore the relationship between various independent variables and the dependent variable, the practice score towards dengue virus prevention. The regression model quantified the individual contributions of each variable to the practice score using both unstandardized (B) and standardized (Beta) coefficients, adjusting for other variables in the model. Additionally, the model included a collinearity test, using tolerance and the Variance Inflation Factor (VIF), to ensure that multicollinearity among predictors did not distort the results. Gender, age, highest education level, place of residence (emirate), employment status, marital status, health insurance status, and smoking status, were independent variables used in the model. Age was treated as a continuous variable. Gender, health insurance, and smoking status were binary indicators. Highest education level, place of residence (emirate), employment status, and marital status were modeled as categorical variables using dummy indicators, with the most prevalent category set as the reference for each.

## Results

**Figure 2.**
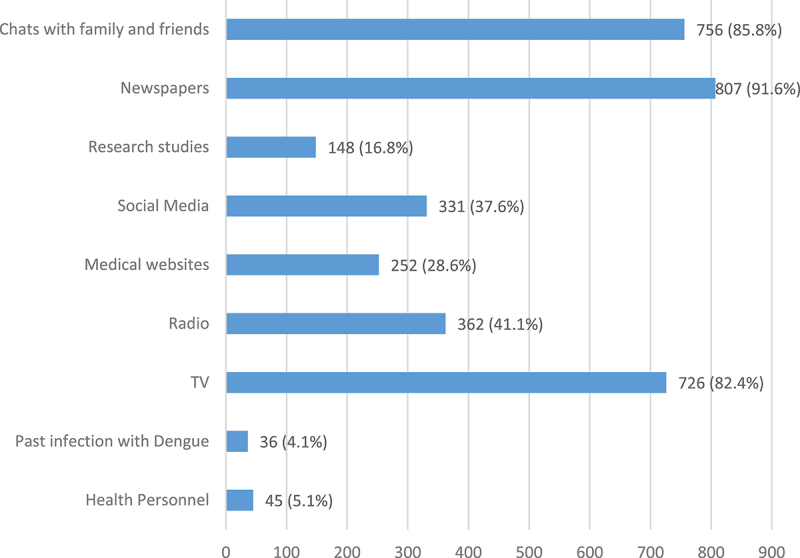
Sources of information about dengue virus (n=1106). The denominator of this item is 881.

In total, 1016 participants completed the questionnaire. Among them, 561 (55.2%) were aged 18-25 years, 727 (71.6%) were female, and 839 (82.6%) lived in Abu Dhabi. Most of the participants were single (583; 52.7%), held undergraduate degrees (639; 57.8%), were employed full-time (648; 58.6%), had health insurance (1060; 95.8%), and had never smoked (687; 62.1%). The demographics of the study participants are presented in Table 1. The top three sources of information about dengue fever, as shown in Figure 2, were newspapers (807; 91.6%), chats with friends and family (756; 85.8%), and television (726; 82.4%).

**Table 1.** Socio-demographic characteristics of the study participants (N=1106).

Parameter	Total, n (%)	Knowledge, median (IQR)	Attitude, median (IQR)
Age (years)			
18-25	561 (50.7%)	11 (10-13)	2 (1-2)
26-35	419 (37.9%)	12 (7-12)	1 (1-1)
36-65	105 (9.5%)	12 (8-16)	2 (1-3)
P value		0.125	0.171
Gender*			
Female	727 (65.7%)	11 (8-12)	1 (1-2)
Male	379 (34.3%)	13 (12-13)	2 (1-2)
P value		**0.001**	0.122
Place of residence			
Abu Dhabi (Capital)	839 (75.9%)	12 (10-13)	1 (1-2)
Dubai	132 (11.9%)	11 (11-11)	2 (2-2)
Sharjah	41 (3.7%)	12 (8-16)	2 (2-3)
Ajman	40 (3.6%)	12.5 (8-16)	2 (2-3)
Fujairah	11(1.0%)	11 (5-16)	1 (1-2)
Ras Al Khaimah	10 (0.9%)	9.5 (2.2-14.7)	1 (1-3)
Umm Al Quwain	6 (0.5%)	10.5 (10.5-15)	2 (2-3)
Western regions	27 (2.4%)	11 (7-18)	1.5 (1.5-3)
P value		0.091	**0.013**
Marital status			
Married	498 (45.0%)	12 (10-12)	1 (1-2)
Single	583 (52.7%)	11 (7-13)	2 (2-2)
Divorced	18 (1.6%)	11.5 (2-14.25)	1 (0-2.25)
Widowed	7 (0.6%)	11 (7-18)	1 (0-3)
Highest education level			
Postgraduate degree	18 (1.6%)	12 (8-15)	2 (1-3)
Undergraduate degree	639 (57.8%)	11 (7-12)	1 (1-2)
High school	316 (28.6%)	7 (7-13)	2 (2-2)
Elementary school	18 (1.6%)	13 (13-18)	1 (1-2)
Uneducated	115 (10.4%)	7 (7-11.5)	1 (1-3)
P value		**0.001**	**0.021**
Employment status			
Full-time employment	648 (58.6%)	11 (9-12)	1 (1-2)
Part-time employment	58 (5.2%)	12 (12-14)	1.5 (1-3)
Self-employed	51 (4.6%)	13 (11-13)	2 (2-2)
Unemployed	337 (30.5%)	11 (7-16)	2 (1-3)
Retired	12 (1.1%)	8 (7-16.75)	1 (0.25-2)
P value		0.123	0.314
Health insurance status			
Yes	1060 (95.8%)	11 (10-13)	1 (1-2)
No	46 (4.2%)	8 (8-13)	1 (1-3)
P value		**0.011**	0.622
Smoking status			
Current smoker	369 (33.4%)	13 (7-13)	2 (1-2)
Former smoker	50 (4.5%)	12 (8-16.25)	2 (1-3)
Never smoked	687 (62.1%)	11 (10-12)	1 (1-2)
P value		0.141	0.239

Differences in median scores were measured using the Mann-Whitney U test and the Kruskal-Wallis H test, as appropriate.

The overall median knowledge score was 11 (IQR: 10-13). Only a minority of participants demonstrated awareness of key aspects of dengue fever; specifically, 24.6% (n=272) correctly identified the disease as a viral infection, and a similar proportion, 24.5% (n=271) recognized that it is transmitted through the bite of infected mosquitoes (Table 2). Additionally, 25.9% (286 participants) acknowledged that high population movements could contribute to the spread of dengue fever. In terms of disease progression and recovery, only 15.7% (n=174) were aware that most individuals recover from dengue within 1-2 weeks, while 32.7% (n=362) understood that second-time infections typically present more severe symptoms. Furthermore, 34.6% (n = 383) recognized that post-recovery fatigue could persist for several weeks, and 37.0% (n = 409) were knowledgeable about the significant reduction in fatality rates with early detection and proper medical care for severe dengue cases. The median knowledge scores were significantly higher among individuals with elementary education (median = 13, IQR: 13-18) and those with postgraduate degrees (median = 12, IQR: 8-15) compared to participants with other education levels (p<0.05). Participants who had health insurance exhibited significantly higher knowledge scores (median = 11, IQR:10-13; p = 0.013) compared to those without insurance (median = 8, IQR: 8-13).

**Table 2.** Knowledge about dengue fever (N= 1106).

Parameter	Total, n (%)
Dengue fever a viral infection [yes]	272 (24.6%)
Dengue fever is transmitted through the bite of infected mosquitoes [yes]	491 (44.4%)
Dengue fever can lead to heart failure or lung problems if not managed properly [yes]	271 (24.5%)
Dengue fever can rarely lead to death [yes]	713 (64.5%)
Most people with dengue have mild or no symptoms [yes]	601 (54.3%)
All mosquitoes found in our environment carry the dengue virus [no]	573 (51.8%)
Mosquitoes actively bite during the day [yes]	722 (65.3%)
Dengue fever is caused by high population movements [yes]	286 (25.9%)
Dengue fever caused by high population density [yes]	822 (74.3%)
Previous infection with Dengue virus (DENV) increases the risk of the individual developing severe dengue fever [yes]	761 (68.8%)
Most people who get dengue fever will get better in 1-2 weeks [yes]	174 (15.7%)
The most common symptoms of dengue fever are high fever, headache, body aches, nausea and rash [yes]	861 (77.8%)
Symptoms that need admission to the hospital are abdominal pain, persistent vomiting, rapid breathing, bleeding gums or nose, fatigue, restlessness, blood in vomit or stool, being very thirsty, pale, and cold skin, and feeling weak [yes]	872 (78.8%)
People who are infected for the second time tend to have more severe symptoms [yes]	362 (32.7%)
After recovery, people who have had dengue may feel tired for several weeks [yes]	383 (34.6%)
There is no specific treatment for dengue fever, the focus is on treating pain symptoms [yes]	846 (76.5%)
Antibiotics are approved to treat dengue fever [no]	464 (42.0%)
Supportive treatment only includes paracetamol [yes]	442 (40.0%)
A vaccine is now available for dengue fever [yes]	817 (73.9%)
Early detection and access to proper medical care greatly lower fatality rates of severe dengue fever [yes]	409 (37.0%)

Only correct responses are displayed.

**Figure 3.**
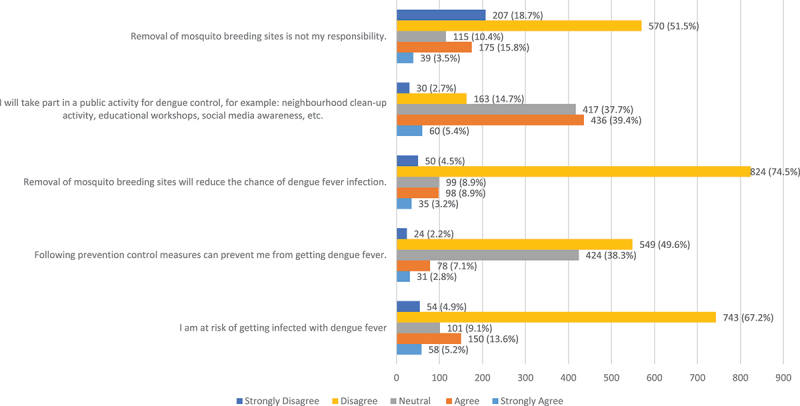
Attitudes towards dengue fever (N=1106).

The overall median attitude score was 1 (IQR: 1-3). Most participants disagreed (n = 824, 74.5%) or strongly disagreed (n = 50, 4.5%) that the removal of mosquito breeding sites would reduce the chance of dengue fever infection (Figure 3). Additionally, 549 (49.6%) and 24 (2.2%) of the participants disagreed or strongly disagreed, respectively, that preventative control measures can protect a person from infection. Furthermore, 743 (67.1%) and 54 (4.9%) of the participants disagreed or strongly disagreed, respectively, that they were at risk of contracting dengue fever infection. Participants who lived in Dubai (median = 2, IQR: 2-2), Ajman (median = 2, IQR: 2-3), Sharjah (median = 2, IQR: 2-3), and Umm Al Quwain (median = 2, IQR: 2-3) showed significantly higher attitude scores compared to participants living in other cities. Those with postgraduate degrees (median = 2, IQR: 1-3) and high school education (median: 2, IQR: 2-2) demonstrated significantly higher attitude scores compared to those with undergraduate degrees (median = 1, IQR: 1-2; *p* = 0.001), those with an elementary school education (median = 1, IQR: 1-2), and those who were uneducated (median = 1, IQR: 1-3).

The overall median practice score was 2 (IQR: 2-2). Approximately half of the participants reported using fans to reduce mosquito presence (n=544, 49.2%) and drinking plenty of fluids if infected with dengue fever (n=538, 48.6%) (Table 3). On the other hand, only 12.6% (n=139) reported eliminating mosquito breeding grounds, and 15.9% (n=176) regularly used insecticide sprays. The use of mosquito repellent creams or sprays while outdoors was particularly low (*n*=44, 4.0%), as was participation in public activities for dengue control (n=59, 5.3%). Measures that involve modifying the physical environment, such as installing mosquito nets for sleeping (n=67, 6.1%), eliminating standing water (n=65, 5.9%), and cutting back bushes (n=74, 6.7%), were also reported by relatively few participants. Similarly, water storage practices, such as covering water containers (n=80, 7.2%) and properly closing them after use (n=84, 7.6%), were adopted by less than 10% of the respondents.

**Table 3.** Dengue fever prevention practices (N=1106).

Parameter	Total, n (%)
I have eliminated mosquito breeding grounds before.	139 (12.6%)
I regularly use insecticide sprayers to kill mosquitoes.	176 (15.9%)
I use insect-repellent creams/liquids/spray while outdoors.	44 (4.0%)
I install mosquito nets for sleeping when there are mosquitoes around.	67 (6.1%)
I took part in public activity for dengue control.	59 (5.3%)
If I am infected with dengue fever, I would drink plenty of fluids.	538 (48.6%)
I will wear bright-colored clothing that covers my body to prevent mosquito bites.	62 (5.6%)
I use fans to reduce mosquitoes.	544 (49.2%)
I eliminate standing water around the house to reduce mosquitoes.	65 (5.9%)
I cut back bushes in the yard to reduce mosquitoes.	74 (6.7%)
I cover water containers at home.	80 (7.2%)
I immediately close water containers after using them.	84 (7.6%)
I properly dispose of items that can collect rainwater on rainy days.	85 (7.7%)
I take mosquito preventive measures before going on a long holiday.	79 (7.1%)
I use mosquito repellent on my body.	68 (6.1%)
I avoid dark areas in the home where there is no light and no wind.	74 (6.7%)

Table 4 presents the results of the regression analysis. Male gender (coefficient = 0.71; CI: 0.428 – 0.993; *p* = 0.001), higher age (coefficient = 0.872, CI: 0.666 0.996; p = 0.001), full-time employment (coefficient = 0.750; CI: 0.598 – 0.902; p = 0.03), and smoking (coefficient = 0.416; CI: 0.253 – 0.579; p = 0.003) were associated with higher practice scores. However, higher education level was associated with a slight decrease in practice scores (coefficient = -0.096; CI: -0.181 – -0.011; *p* = 0.028). The Variance Inflation Factor (VIF) and tolerance values for all independent variables were measured. VIF values ranged from 1.059 to 1.871, and all tolerance values were above 0.5. These results fell within accepted thresholds, with VIF values below 10 and tolerance values above 0.1, indicating that multicollinearity was not a concern.

**Table 4.** Predictors of good dengue fever prevention practices.

Independent variables included in the model	Unstandardized B	95% CI (lower-upper)		*p*	Collinearity	
		Lower	Upper		Tolerance	VIF
Gender	0.711	0.428	0.993	**0.001**	0.840	1.191
Age	0.872	0.666	0.996	**0.001**	0.764	1.309
Highest education level	-0.096	-0.181	-0.011	**0.028**	0.869	1.151
Place of residence	0.525	0.426	1.624	0.261	0.789	1.267
Employment status	0.750	0.598	0.902	**0.03**	0.567	1.763
Marital status	0.014	-0.286	0.314	0.926	0.534	1.871
Health insurance status	-0.259	-0.892	0.375	0.423	0.945	1.059
Smoking status	0.416	0.253	0.579	**0.003**	0.650	1.539

Bold indicates significant results. VIF: variance inflation factor. CI: confidence interval.

## Discussion

The present study found a concerning lack of knowledge about dengue fever among the UAE public. Specifically, a significant proportion of participants had gaps in their understanding of key aspects of the disease, including its etiology, transmission, and clinical course. Our findings are consistent with a study conducted in Nepal, which also reported poor public knowledge about dengue fever [23]. In contrast, studies from Saudi Arabia [24], Australia [25], Yemen [26], and India [27] reported a higher level of public knowledge about dengue fever. Notably, a study from Jordan demonstrated satisfactory levels of knowledge regarding the transmission and common symptoms of another viral infection, COVID-19 [28]. This suggests that public understanding may be more robust for more recent or highly publicized outbreaks, potentially due to greater focus on public health campaigns. These variations might be attributed to differences in public health initiatives, the frequency and recency of dengue outbreaks, and the scope of targeted education campaigns in these regions. Additionally, our findings showed disparities in knowledge levels based on educational background and health insurance status, which provide insights into the demographic factors affecting health literacy. Individuals with either elementary education or postgraduate degrees had significantly higher knowledge scores, suggesting that the extent and quality of education considerably impact health awareness. Conversely, lower knowledge scores among those without health insurance may reflect limited access to healthcare services, where such information is typically disseminated. Similarly, in Saudi Arabia, education level and employment status have been found to be significantly associated with participants’ knowledge of dengue fever [24].

Our results identified newspapers, chats with friends and family, and television as UAE participants’ top three sources of information about dengue fever. Similar studies from various countries have also shown that television and radio are significant channels for disseminating dengue-related information. For instance, in Bangladesh, 44.2% of respondents identified television as their primary source of information, followed by friends and family (26.8%) and newspapers (13.2%) [29]. Similar patterns were observed in Pakistan and Nepal, where television and radio were the main channels, with significant reliance also on interpersonal sources like friends and family [30]. The reliance on traditional and interpersonal sources highlights a gap in the utilization use of direct health professional advice which is critical for ensuring the accuracy of health information. While interpersonal networks provide readily accessible information, the quality and accuracy of such information can vary, potentially undermining public health responses and individual health behaviors.

The present study indicated unfavorable attitudes towards dengue fever prevention strategies and the effectiveness of control measures among the UAE public. This is consistent with findings from a previous study in Malaysia, where more than half of the participants reported negative attitudes toward dengue fever [31]. A notable example of an unfavorable attitude in our study was that a significant proportion of participants disagreed with the notion that they were at risk of contracting dengue. This suggests a low perceived susceptibility and severity regarding dengue in the UAE. In contrast, a study in the Philippines found that a higher perceived risk among educators was associated with better prevention practices [32]. In regions where outbreaks are frequent or have recently been severe, public perception tends to align more closely with the actual risk [32]. We re-audited our dataset and coding to ensure that the results accurately represent participant responses, as disagreement over the protective benefit of breeding-site removal is rare in dengue KAP literature (i.e., no reverse coding or tabulation errors). There were likely several contextual factors at play. First, considering the UAE’s traditionally non-endemic status and recent increases associated with unusual rainfall or floods, perceived susceptibility to dengue may be relatively low; this reduced perceived risk might weaken attitudes toward prevention. Second, high-profile official vector-control initiatives might shift the focus from households to authorities, thereby undermining support for reducing household sources [33]. Third, participants reported that they primarily relied on interpersonal networks and the media for information; previous research indicates that media exposure does not always result in accurate beliefs about specific source-reduction strategies in the absence of targeted reinforcement by health professionals [32]. Lastly, home-container breeding may be less apparent to residents in metropolitan areas where apartments predominate, which would further weaken support for household source-reduction efforts [31,34]. This pattern is also consistent with evidence that perceived risk mediates the pathway from knowledge to preventive practice; when perceived susceptibility is low, confidence in personal preventive measures likewise declines [35]

In our study, attitudes were significantly associated with place of residence and education level, suggesting that these socio-demographic factors play a critical role in shaping attitudes towards disease prevention. Similarly, in Central Nepal, attitudes towards dengue were found to be influenced by demographic factors such as education and income [23]. Higher socio-economic status is often correlated with better knowledge and more proactive attitudes towards dengue prevention, likely due to better access to resources and health education. These patterns are in line with studies showing that higher socioeconomic position (for example, higher education, income, or employment) is associated with greater dengue-related knowledge and more favorable attitudes [10,31]. The UAE sample is concentrated in large, highly urbanized emirates where high-rise apartments dominate the housing stock (e.g., apartments comprised ~82% of Dubai residential transactions in 2024–25) [36]. These types of homes might reduce support for source-reduction initiatives. Simultaneously, the country experienced abnormally high rainfall in April 2024, after which officials reported the elimination of nearly 400 breeding sites. The attitudes and behaviors we observed are likely influenced by these urban and climatic conditions. The necessity of household-level measures, even in apartments, is further supported by the fact that Aedes aegypti is a container-breeding mosquito and can use artificial water-holding containers in and around residences, including high-rise apartments, such as domestic water-storage tanks/drums and smaller containers like flower pots and other receptacles that can hold standing water indoors (e.g., within apartments) or outdoors [37,38]The findings of this study revealed poor dengue prevention practices among participants. The low rates of mosquito breeding site elimination, insecticide use, and mosquito repellent application are particularly concerning, as these practices are fundamental to dengue prevention. Their underutilization suggests a significant gap in knowledge or behavior that needs to be addressed. The low prevalence of environmental modifications, such as installing mosquito nets, eliminating standing water, and cutting back bushes, is also noteworthy. These practices are effective in reducing mosquito populations and should be more vigorously promoted. Additionally, the limited adoption of water storage practices, like covering and properly closing water containers, is particularly concerning given the crucial role these containers play as mosquito breeding sites.

In this study, demographic factors such as gender, age, employment status, smoking habits, and education levels were significantly associated with dengue prevention practices. The finding that males had higher practice scores than females may be related to gender-specific roles or activities that expose them more frequently to outdoor environments where preventive measures are more salient or necessary. This is somewhat counterintuitive, as studies on women’s participation in dengue prevention, often highlight that women are typically more engaged in household preventive practices due to their roles within the family and community [39]. A study in Cambodia found that males were more likely to seek medical attention when suspecting dengue infection. Further research is needed to explore the relationship between sociodemographic factors and dengue prevention practices [40].

Interestingly, current smokers reported higher practice scores, which may seem paradoxical given the generally negative association between smoking and health behaviors. A similar pattern was observed in a study from Bangladesh, where individuals in urban groups at high risk of cholera showed unexpectedly strong adherence to cholera prevention measures [41]. Despite poor infrastructure and lower overall health knowledge, these groups demonstrated good use of oral rehydration solutions and responded well to vaccination efforts [41]. Nevertheless, this finding warrants further investigation. This finding may be influenced by confounding factors or reflect a complex interplay of behaviors and attitudes within the study population.

The current findings also showed a slight decrease in practice scores with higher education levels, suggesting a discrepancy between knowledge and action. While higher education levels often correlate with greater knowledge, it does not always translate into practice [40,42]. This gap could be attributed to various factors, such as perceived barriers, skepticism about the effectiveness of preventive measures, or a mismatch between educational messages and their practical applicability in daily life [43]. For example, a study found that university students, despite having knowledge about self-regulated learning strategies, often failed to apply them in practice. The students cited reasons such as the perceived irrelevance of the strategies to specific tasks, the effort required, and doubts about their effectiveness in real-life scenarios [44].

Based on our findings, three implementation priorities emerge. First, risk communication to raise perceived susceptibility: Our data show low perceived risk and skepticism toward household source reduction. Authorities can deploy WHO’s dengue RCCE toolkit to deliver localized, plain-language messaging that links post-rainfall standing water in specific urban settings (e.g., balcony planters, roof tanks, gully traps) to infection risk, and uses trusted mass media plus building-level campaigns to shift norms [45]. Second, apartment-focused source reduction: Partner with municipalities, building managers, and owners’ associations to institutionalize routine inspections (drains, tanks, condensate trays), ensure maintenance, and provide resident checklists. Third, adopt communication-for-behavioural-impact (COMBI) methods that set specific, measurable household actions (e.g., weekly 10-minute “dry day” checks), track adoption, and leverage interpersonal networks identified in our sample [46].

## Study limitations

The survey was distributed using convenience sampling, which may not have yielded a representative sample of the UAE population. This non-random sampling method could have resulted in the overrepresentation or underrepresentation of certain groups, potentially skewing the results and limiting the generalizability of the findings. Additionally, relying on online distribution channels may have disproportionately attracted younger, more tech-savvy participants, reducing the applicability of our findings to older or less digitally engaged individuals. Furthermore, as the UAE hosts a large expatriate population, there is a concern that the study findings might not accurately reflect the knowledge, attitudes, and practices of the permanent resident population. The data, obtained using a convenience sampling method, exhibited deviations from normality despite efforts to ensure appropriate model assumptions. Although multiple linear regression is often robust to minor departures from normality, such variations may nevertheless impact the accuracy of our findings. Furthermore, our sample selection method was non-random, which limits the external validity of the findings and may diminish the generalizability of our conclusions. To accurately represent the target population, future research should consider using probability sampling approaches, and the results should be assessed judiciously. We did not capture nationality/residency (expatriate vs. Emirati) status, which limits subgroup interpretation in a country where expatriates constitute the large majority of residents. Future studies should stratify by residency status and emirate. Compared with the national population, where males outnumber females and working-age groups predominate, our sample (71.6% female; 55.2% aged 18–25) over-represents young women. It is also concentrated in Abu Dhabi (82.6%) relative to the national distribution across emirates. These imbalances likely bias KAP estimates toward views of younger, female, Abu Dhabi–based residents. Finally, our quantitative findings point to low perceived susceptibility and skepticism toward household source reduction, yet they do not reveal underlying drivers. Qualitative interviews or focus groups with residents (e.g., high-rise tenants, building managers, outdoor workers) would help unpack beliefs about locus of responsibility, perceived efficacy of actions in apartments, and post-flood experiences

On the other hand, to our knowledge, this is among the first large, multi-emirate KAP datasets on dengue in the UAE general population (N = 1106). It (a) documents an atypically low endorsement of household source-reduction effectiveness, contrary to the prevailing international KAP pattern, (b) details under-utilization of specific preventive practices relevant to high-rise urban living, and (c) links information channels and sociodemographic factors to KAP domains. These granular findings directly inform emirate-level RCCE and building-focused source-reduction strategies.

## Conclusion

This study showed significant gaps in the knowledge, attitudes, and practices regarding dengue fever prevention among the UAE population. Despite the use of multiple linear regression and other analytical techniques, non-random sampling and departures from normalcy may affect the findings’ generalisability, therefore they should be interpreted cautiously. The implementation of focused, evidence-based education campaigns and community engagement programs aimed at raising public awareness of dengue transmission, promoting the removal of mosquito breeding sites, and encouraging the use of preventive measures like insecticide sprays and mosquito repellents are among the recommendations arising from these findings. Furthermore, using reliable information sources like newspapers, television, and personal networks to disseminate accurate and locally relevant advice may increase public receptivity and ultimately lead to more successful prevention initiatives and a lower incidence of dengue in the UAE.

## Data Availability

The data that support the findings of this study are available from the corresponding author upon reasonable request.
